# High Shear Conversion of Elemental Bismuth in Water Under Air to 2D Bismuth Oxycarbonate

**DOI:** 10.1002/chem.202502202

**Published:** 2025-09-10

**Authors:** Fayed Abdullah Alrashaidi, Jason R. Gascooke, Mohammed Z. Asiri, Abigail K. Mann, Ashley Slattery, Jonathan A. Campbell, Youhong Tang, Colin L. Raston

**Affiliations:** ^1^ Institute for Nanoscale Science and Technology College of Science and Engineering Flinders University Bedford Park Adelaide South Australia 5042 Australia; ^2^ Department of Chemistry College of Science Jouf University P.O. Box 2014 Sakaka 72388 Saudi Arabia; ^3^ School of Physics Chemistry and Earth Sciences University of Adelaide Adelaide South Australia 5005 Australia; ^4^ Physics Department Prince Sattam Bin Abdulaziz University Al‐Kharj 16278 Saudi Arabia; ^5^ Adelaide Microscopy The University of Adelaide Adelaide South Australia 5005 Australia

**Keywords:** 2D bismuth oxycarbonate, carbon dioxide capture, green chemistry, high shear, vortex fluidic device

## Abstract

2D Bismuth oxycarbonate (2D BOC) nanosheets have a unique layered structure with many applications, including capture and reduction of carbon dioxide. Processing powdered elemental bismuth in water under ambient air conditions using a vortex fluidic device (VFD) results in the formation of 2D BOC without the need of surfactants or other excipients. The induced high shear mechanical energy in the form of micron/submicron topological typhoon like spinning top (ST) fluid flow drives the conversion, which we propose initially melts the metal particles which are spontaneously oxidised at the liquid‐quartz tube interface to form 2D bismuth oxide (Bi_2_O_3_). Then it reacts with atmospheric CO_2_ to form 2D BOC. These sheets, ≤ 15.7 nm thick, are in the orthorhombic phase with a lattice spacing of 0.29 nm, which is converted to Bi_2_O_3_ monoclinic phase as an exothermic process at 269 °C.

## Graphical abstract

Powdered elemental bismuth in water forms 2D bismuth oxycarbonate nanosheets under high shear in a rapidly rotating tube housed in a vortex fluidic device, without the need of surfactants or other excipients, with uptake of atmospheric carbon dioxide.

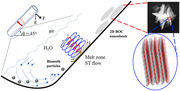

## 1 Introduction

Two‐dimensional nanomaterials (2D NMs) are described as ultra‐thin sheets down to one atom thick and can be prepared from bulk material. The scientific interest in 2D NMs stems from the discovery in 2004 of the exfoliation of graphite into graphene 2D carbon layers. Since then, research has intensified to discover other 2D NMs, driven by their unique and potentially advantageous properties for application in, for example, electronics, energy, and catalysis. One class of these NMs is 2D pnictogens, namely phosphorene, antimonene, and bismuthene.^[^ ^1^ ^]^ For the latter, there is also interest in forming 2D bismuth oxide (Bi_2_O_3_) and related 2D compounds. Recently, 2D Bi‐based compounds have attracted considerable interest due to their distinctive layered structures with potential in photocatalysis and environmental applications. 2D bismuth oxycarbonate (BOC), Bi_2_O_2_CO_3_, also known as bismutite or bismuth sub‐carbonate, was discovered by Grice et. al in 2002.^[^ ^2^ ^]^

2D BOC stands out for promoting photocatalytic reactions under visible light, being effective for breaking down organic contaminants/pollutants and reducing carbon dioxide (CO_2_) emissions in an environmentally friendly way.^[^ ^3^ ^]^ 2D BOC is also of interest as a semiconductor, having a band gap of 3.1–3.5 eV.^[^ ^4^, ^5^ ^]^ It has a unique stacking arrangement comprised of alternating layers of (Bi_2_O_2_)^2+^ interleaved with CO_3_^2−^, having high chemical stability and properties suitable for both optical and electrocatalytic applications. Indeed, the material is selective toward CO_2_ reduction, in forming formate/formic acid at low overpotentials.^[^ ^6^, ^7^, ^8^, ^9^, ^10^ ^]^ Also noteworthy is that the use of 2D BOC aligns with the principles of green chemistry, in that bismuth is a “green” heavy metal.

Several methods have been used to fabricate 2D BOC nanosheets including electrochemical and chemical synthesis,^[^ ^11^, ^12^ ^]^ hydrothermal,^[^ ^13^ ^]^ and solvothermal processing.^[^ ^14^ ^]^ Limitations of such methods include the use of templates involving polyvinylpyrrolidone, and sodium dodecyl sulfate (SDS), and associated lengthy processing times^[^ ^5^ ^]^ as well as difficulties in scaling up the synthesis for commercial applications. Inadequate removal of the templates can result in impurities in the material.^[^ ^4^, ^15^, ^16^ ^]^ Few‐layer BOC can be prepared electrochemically involving exfoliation of bulk Bi metal in the presence of excipients such as aqueous potassium chloride (KCl) and sodium carbonate (Na_2_CO_3_).^[^ ^17^ ^]^ Na^+^ ions facilitate exfoliation of bismuthene, acting as an intercalating agent, but this compromises the efficiency of the process and the purity of the isolated BOC.^[^ ^6^ ^]^ Moreover, the synthesis of 2D BOC has high energy consumption requirements which is associated with discharges in dielectric liquids.^[^ ^14^, ^18^ ^]^ There are also challenges in scaling up the synthesis of 2D BOC as well as improving the green chemistry metrics of the process for translating into industry, all of which are addressed in the present work.

**Figure 1:**
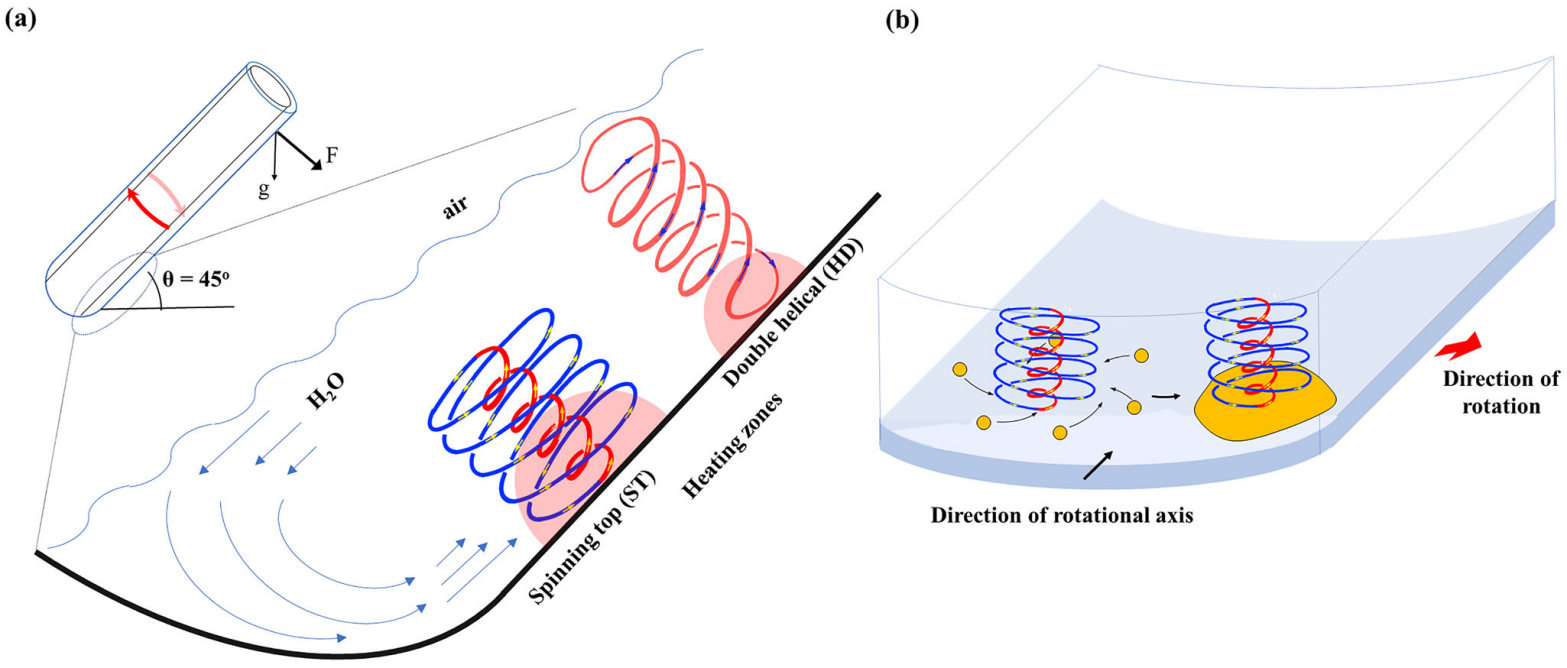
a) Schematic diagram illustrating the high shear topological fluid flow in the confined mode of operation of the VFD, with the b) transformation of the elemental Bi particles at the base of the spinning top (ST) flows interfacing with the surface of the quartz tube.

We have developed a more benign method to directly address the above limitations for preparing 2D BOC nanosheets. This approach involves the use of a vortex fluidic device (VFD), Figure 1, in generating pristine 2D BOC which eliminates the need for adding any excipients to the water used in the processing, while also having the potential for scaling up the process for downstream applications. A standard VFD houses a quartz tube 18.5 cm length, 17.5 mm internal diameter (ID), and 20 mm outer dimeter (OD), which is tilted (*θ*) at 45° and rapidly rotated, typically up to a rotational speed (*ω*) of 9k rpm. The *θ* = 45° has been proven as the optimal value for a diversity of applications including for nanocarbon and inorganic material fabrication. It was fixed as such in the present work.^[^ ^19^, ^20^, ^21^, ^22^ ^]^ The VFD is effective in exfoliating preformed 2D NMs, as in graphene and graphene oxide from graphite,^[^ ^23^ ^]^ phosphorene nanosheets,^[^ ^24^ ^]^ 2D boron nitride,^[^ ^25^ ^]^ MXene,^[^ ^26^ ^]^ and MoS_2_.^[^ ^27^ ^]^ In addition, it is effective in transforming material in situ for preparing 2D material, melting and recrystallizing 2D antimonene,^[^ ^28^ ^]^ transforming liquid gallium and gallium/indium eutectic melts into 2D gallium oxide sheets,^[^ ^29^ ^]^ and 2D gallium and indium composites,^[^ ^30^ ^]^ respectively. The present research using the VFD involves in situ melting of elemental Bi and reaction with CO_2_, affording 2D BOC, with its formation understandood mechanistically.

The VFD is a thin film microfluidic platform where micron/submicron topological fluid flows impart mechanical energy, depending on the rotational speed, *ω*, of a tube tilted relative to the horizontal position, defined as the tilt angle, *θ*, temperature, and properties of the liquid. The high shear topological fluid flows are the spinning top (ST) (typhoon like) flow arising from the Coriolis force from the hemispherical base of the tube, and double helical (DH) flow as a side wall Coriolis force twisting Faraday wave eddies, Figure 1(a).^[^ ^19^, ^31^, ^32^ ^]^ The temperature generated by localized heating in the ST exceeds the melting point of antimony (630 °C) in an organic solvent,^[^ ^28^ ^]^ with rapid heat dissipation into the bulk liquid and the surroundings ensuring that temperature fluctuations for the liquid is minimal, and in this context the VFD is effective in carrying out extremely exothermic reactions at room temperature. Also noteworthy is that the process of determining the nature of the ST high shear topological flow as a result of Coriolis forces from the hemispherical base of the tube involved melting and crystallization of elemental Bi (m.p. of 271.4 °C) in an organic solvent.^[^ ^19^ ^]^

## 2 Experimental Section

### 2.1 Materials

Bismuth powder, 100‐mesh, 99% purity, was purchased from Sigma Aldrich, Australia, and Milli‐Q water was used in all experiments.

### 2.2 Synthesis of 2D BOC Nanosheets

**Figure 2:**
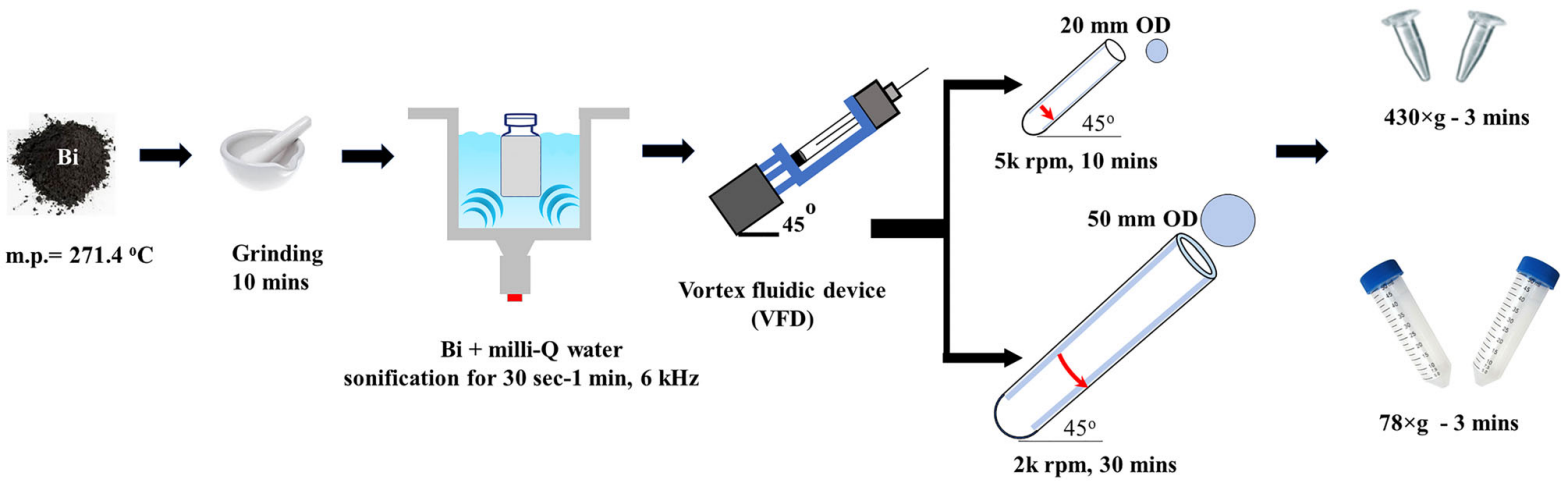
Schematic diagram for the fabrication 2D BOC nanosheets at room temperature in the VFD using the standard 20 mm OD quartz tube, and the upsized 50 mm OD quartz tube, followed by centrifugation to remove any starting material.

Bi powder was ground for 10 minutes (mins) in an air atmosphere using a mortar and pestle, then dispersed in Milli‐Q water at concentrations of 0.5, 1, and 2 mg.mL^−1^ followed by sonification for 30 to 60 seconds (sec) at 6 kHz to generate stable colloidal suspensions, Figure S2 in Supporting Information. Then 2 mL of the mixture was added to the VFD quartz tube (18.5 mm in length, 17.5 ID) and the device was operated in the confined mode for 10 minutes with *ω* = 5k rpm in an air atmosphere at room temperature. The liquid was then centrifuged for 3 minutes (430 × g) to precipitate unprocessed bulk Bi, Figure 2. A scaled‐up liquid was then centrifuged for 3 minutes (78 × g) to precipitate unprocessed bulk Bi, Figure 2. The scaled up synthesis was performed for 20 mL of 3 mg.mL^−1^ sample, with the liquid processed in an up‐sized large VFD (L‐VFD)^[^ ^33^ ^]^ housing a 45 cm long quartz tube with an OD of 50 mm and an ID of 45 mm, which was rotated at *ω* = 2k rpm for 30 minutes. The L‐VFD tube was also tilted at *θ* = 45° which is optimal for a number of applications of VFD devices in general.^[^ ^19^, ^33^ ^]^ The supernatant was drop‐cast onto a silicon (Si) wafer for characterization using scanning electron microscopy (SEM), X‐ray photoelectron spectroscopy (XPS), atomic force microscopy (AFM), Raman spectroscopy, and X‐ray diffraction (XRD) Additionally, the materials isolated using the L‐VFD were utilized for further characterization using simultaneous thermal analyzer (STA).

### 2.3 Characterization

SEM characterized used a FEI Inspect F50 instrument. Size distribution plots were created via Fiji ImageJ software. AFM images were acquired using Multimode 8 AFM with Mikromasch HQ: NSC15 Si probes (nominal tip radius 8 nm) and Nanoscope V controller, using the tapping mode in an ambient atmosphere. Sensitivity and spring constant (21–35 Nm^−1^) were calibrated following methods by Sader et al.^[^ ^34^ ^]^ Setpoint, scan rate and feedback gains were optimized, and the set point was maintained at 80% to 90%. XRD using a Buker D8 Advance Eco diffractometer with Bragg–Brentano geometry using a Co Kα source, *λ* = 1.7889 Å. Scanning transmission electron microscopy (STEM) images were taken using a CS‐corrected Thermo‐Scientific Titan microscope operating at 200 kV. Raman spectra were obtained using a Horiba Scientific XplorRA confocal Raman microscope with three different laser excitation wavelengths, 532, 638, and 786 nm. Spectra were calibrated utilizing the 520 cm^−1^ (Si) peak and were obtained with a 50 × objective with a numerical aperture of 0.55. X‐ray photoelectron spectroscopy (XPS) was performed with a base pressure of a few 10^−9^ mbar using an ultrahigh‐vacuum device constructed by SPECS. Experiments were conducted with a UHV nonmonochromatic X‐ray source with a Mg anode producing 1254.6 eV photons. Thermogravimetric analysis (TGA) and differential scanning calorimetry (DSC) measurements used a 449 F5 Jupiter STA with nitrogen injected into the furnace at 20 mL.min^−1^, for a ∼ 5 mg sample size prepared in the L‐VFD. The temperature was increased at 10 °C.min^−1^, from 60 to 600 °C.

## 3 Results and Discussion

**Figure 3:**
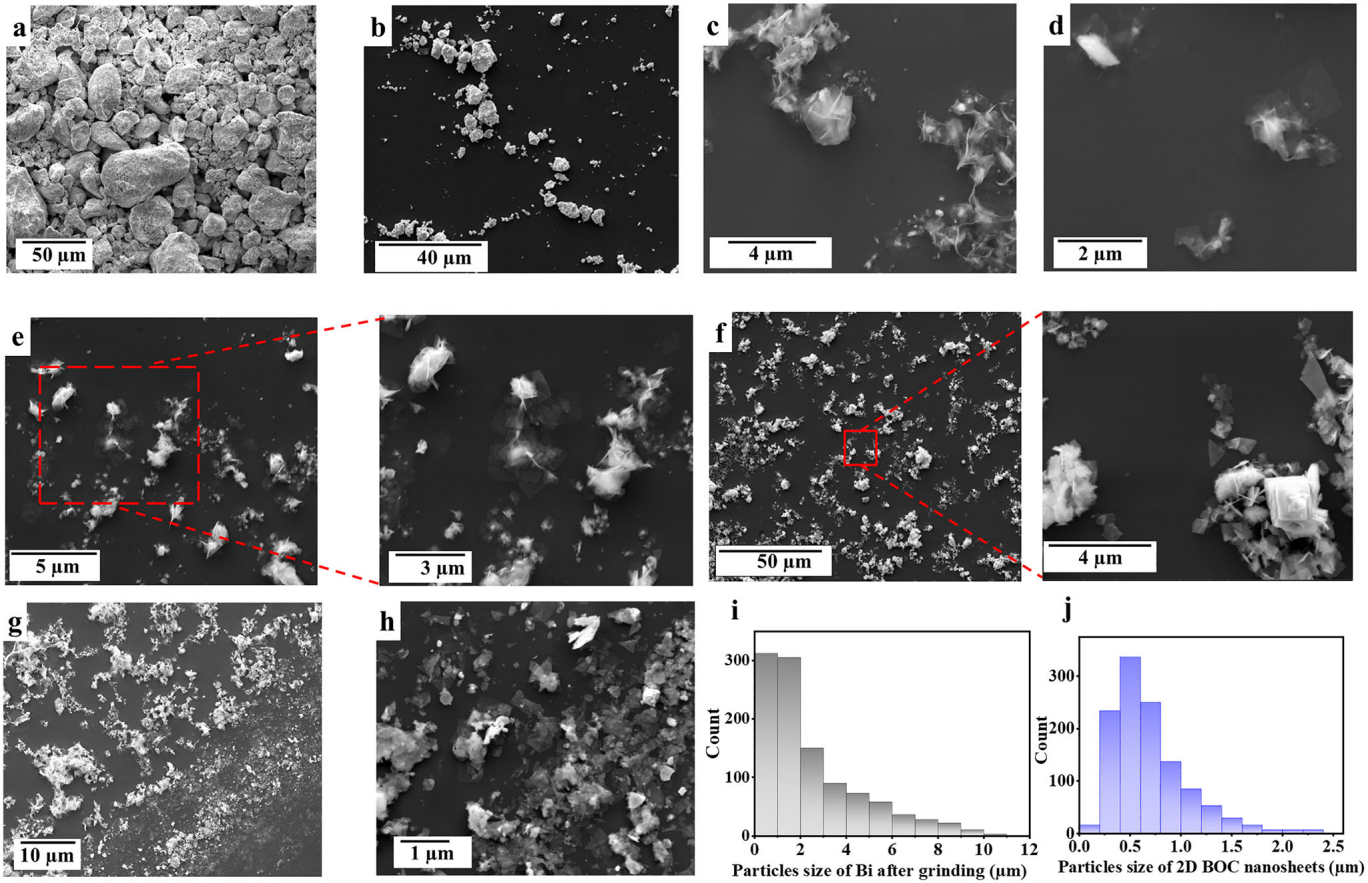
SEM images of Bi as received a), b) Bi particles after grinding in air using a mortar and pestle, c–h) 2D BOC sheets after processing in the VFD at *ω* = 5k rpm, *θ* = 45°, in air atmosphere for 10 minutes at different concentrations of the material suspended in water, c), d) 0.5 mg.mL^−1^, e) 1 mg.mL^−1^, f) 2 mg.mL^−1^, and g), h) 3 mg.mL^−1^ processed in the up‐sized 50 mm OD tube at *ω* = 2k rpm under an atmosphere of air for 30 minutes, then drop cast on a Si wafer. i) Particle size distribution of Bi as received after grinding, and j) lateral dimension particle size distribution of 2D BOC nanosheets.

SEM images were taken to investigate the morphology before and after processing aqueous suspensions of powdered Bi in the VFD, Figure 3; the as received Bi powder had a mean size of ∼ 50 µm, Figure 3(a), with the mean size of the powder after grinding at 2 to 20 µm, Figure 3(b). Suspensions of the latter in water at 0.5, 1, and 2 mg.mL^−1^ were sonicated at 6 kHz for 30 to 60 seconds prior to processing in the 20 mm OD VFD tube for 10 minutes, *θ* = 45°, *ω* = 5k rpm Figure 3(c–f). SEM images of material generated during 30 minutes in the L‐VFD for Bi at 3 mg.mL^−1^, *θ* = 45° and *ω* = 2k rpm are presented in (Figure 3g,h), for which the isolated yield was ∼ 28%, Equation S4. The larger particles were removed by centrifugation at 430 × g for 3 minutes for samples processed in the 20 mm OD quartz VFD tube, and 78 × g for 3 minutes for samples processed in the L‐VFD.

Processing Bi suspensions in a 20 mm VFD quartz tube, *ω* = 5k rpm, *θ* = 45°, results in 2D nanomaterials. This is consistent with its formation occurring at the base of the ST flows which are micron to submicron in diameter. The localized heating for this topological fluid flow is at the interface of the quartz tube with the liquid. This topological fluid flow has been identified as effective in forming 2D nanomaterial in general in the VFD, and more recently in twisting coiled toroids of single wall carbon nanotubes (SWCNTs) into either left‐ handed or right‐ handed Figure of 8s (lemniscates).^[^ ^35^ ^]^ Water in the tube within the ST flow is forced down onto the surface of the tube, with the high heat friction melting the Bi which we propose is then spontaneously oxidized to form 2D Bi_2_O_3_. In the presence of CO_2_ from air, it is converted to 2D BOC nanomaterial sheets 1 to 3 µm in cross section. The 2D material is deposited at the base of the ST flows which are generated at the hemispherical base of the tube and then move across the surface of the tube toward its top. The effect of changing the concentrations of suspensions of Bi powder on the synthesis of 2D BOC is shown in (Figure 3c–h). The bulk Bi pre‐VFD processing has a mean diameter ∼ 2.52 µm and cross‐sectional area of ∼ 5.61 µm^2^, Figure 3(i) and (Figure S1a). The size distribution of 2D flat BOC nanosheets formed from a 2 mg.mL^−1^ Bi suspension processed in the VFD had a mean diameter of ∼ 0.69 µm and cross‐sectional area of 0.24 µm^2^, Figure 3(j) and Figure S1(b). Hence, the SEM images are consistent with the fabrication of 2D flat BOC nanosheets after processing elemental Bi in the VFD in 10 minutes using water as a solvent.

**Figure 4:**
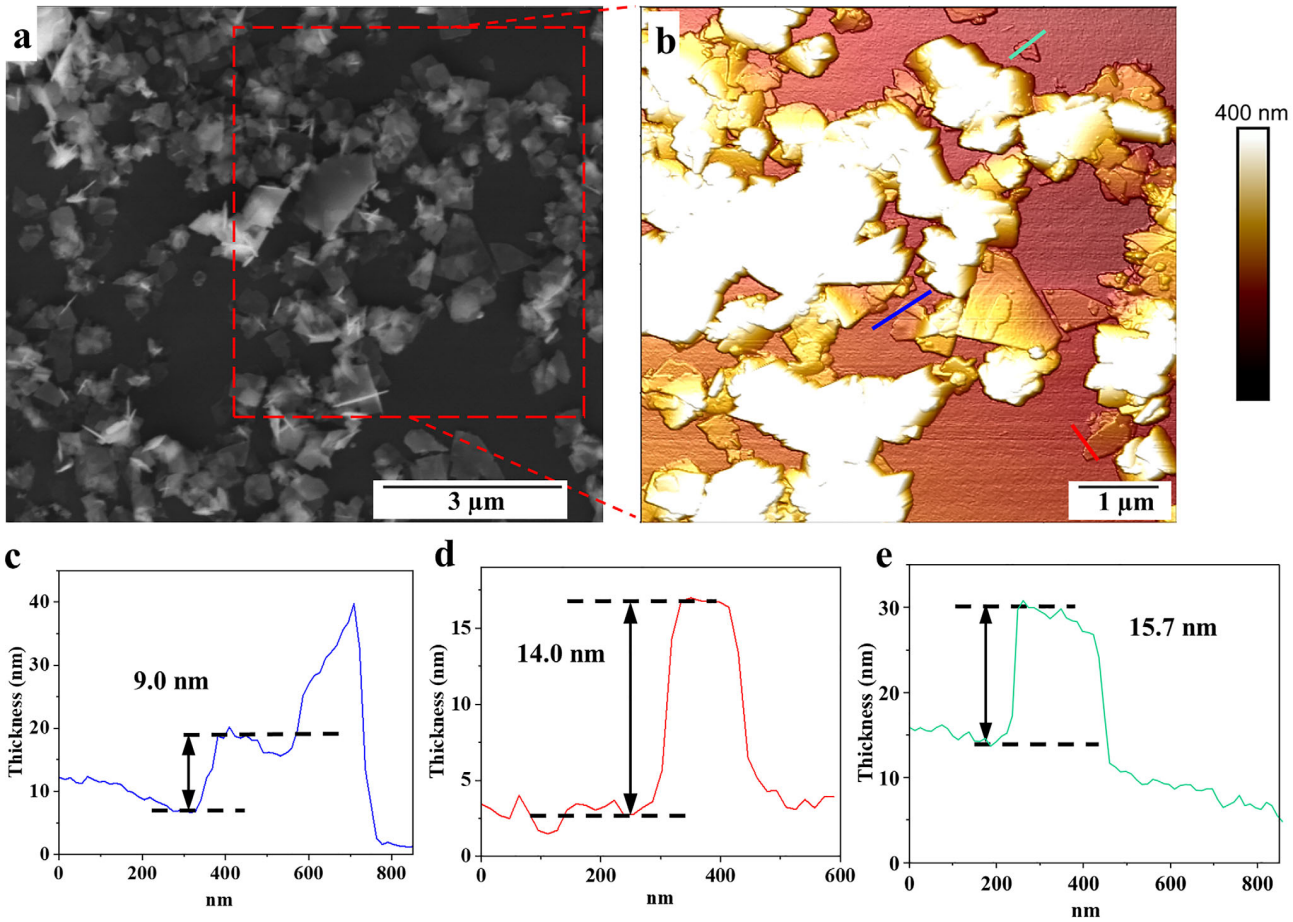
a) SEM image of 2D BOC nanosheets, b) 3D AFM image of 2D BOC nanosheets fabricated in water at room temperature, 10 minutes processing in a standard VFD 20 mm OD, *ω* = 5k rpm, *θ* = 45°, in confined mode, concentration 1 mg. mL^−1^, drop cast on a Si wafer, c–e) height profiles for the indicated positions.

AFM measurements were used to determine the thickness of 2D BOC sheets that were synthesized using a 20 mm VFD quartz tube, Figure 4. The thickness of different agglomerated sheets examined were between 9 to 15.7 nm. The AFM images show that the sheets are flat and thin and stack together with variable thickness across the layers. These results confirm that the VFD processing fabricates 2D nanosheets which aligns with the SEM results.

**Figure 5:**
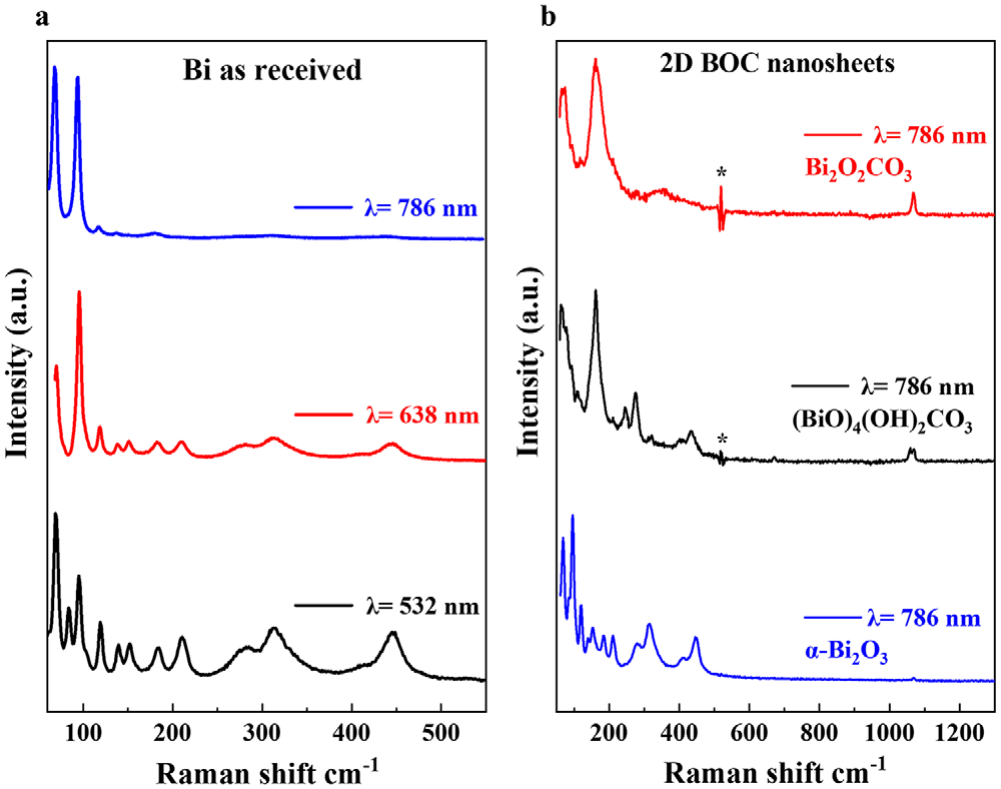
a) Raman spectra of as received Bi, b) and of 2D BOC nanosheets fabricated in water at room temperature, 10 mins processing in a standard VFD 20 mm OD, *ω* = 5k rpm, *θ* = 45°, confined mode, concentration 1 mg. mL^−1^, drop cast on a Si wafer. The background Si spectrum has been subtracted and the features marked with (*) indicate an artefact resulting from this process.

Raman spectra of Bi as received and following VFD processing are shown in (Figure 5a,b). The Raman spectra of bulk Bi was investigated using three different laser excitation wavelengths, 532, 638, and 786 nm, which revealed notable differences in spectral features. This is in accordance with that reported by Trentelman^[^ ^36^ ^]^ where Raman signals from the surface oxidation layer becomes enhanced at lower wavelengths due to absorption in the visible region. When excited with the 786 nm laser, the spectrum predominantly displays sharp and well‐defined peaks at ∼70 and ∼94 cm^−1^ corresponding to the *E*_g_ and *A*_1g_ phonon modes in metallic bismuth, respectively. In contrast, spectra acquired with 638 and 532 nm lasers exhibit additional bands and broader features, which can be attributed to the presence of Bi_2_O_3_ layers formed on the sample surface.^[^ ^36^ ^]^ These additional peaks at 84, 119, 139, 153, 185, 210, 284, 313, and 447 cm^−1^ are consistent with that reported previously for α‐Bi_2_O_3_.^[^ ^36^, ^37^ ^]^ This comparative analysis underscores the importance of laser wavelength selection in Raman spectroscopy for distinguishing between bulk and surface states in Bi‐containing samples. Thus, Raman spectra of the VFD‐processed material were performed using the 786 nm laser.

Raman spectra of a processed sample recorded with a 786 nm excitation laser is shown in Figure 5(b). Several different spectra were obtained, depending on the location sampled, and thus representative spectra are shown. The upper spectrum in Figure 5(b) is attributable to BOC, having bands at ∼ 74, 162, 350, and 1068 cm^−1^, as previously reported.^[^ ^38^, ^39^, ^40^, ^41^ ^]^ The bands below 350 cm^−1^ are assigned as BOC lattice vibrational modes, and the 1068 cm^−1^ peak is assigned to the ν_1_ symmetric stretch of carbonate, CO_3_^2−^.^[^ ^38^ ^]^ The Raman spectrum in (Figure 5b) was very similar to that observed by Taylor et al. for which the authors assign as for the basic bismuth carbonate, (BiO)_4_(OH)_2_CO_3_.^[^ ^38^ ^]^ Extra peaks appear in the spectrum at 247 and 276 cm^−1^ that have no corresponding partner in the Bi_2_O_2_CO_3_ spectrum. The spectra artefacts following subtraction of the Si spectrum are apparent at 521 cm^−1^ due to noise when removing the intense Si peak which is ∼200 times more intense than the baseline signal. These have tentatively been assigned as concerted motion of the OH^−^.^[^ ^38^ ^]^ Finally, Raman spectra of α‐Bi_2_O_3_ were observed (lower trace in Figure 5(b)) indicating that not all of the metal oxide formed during VFD processing was fully converted to 2D BOC by uptake of atmospheric CO_2_ which is constant with a small amount of α‐Bi_2_O_3_ observed in the XRD patterns. Overall, the use of a 786 nm laser for Raman excitation allows for clear identification of the vibrational modes of Bi_2_O_3_ and carbonates and supports the formation of 2D BOC nanosheets during VFD processing in water for 10 minutes at room temperature in air, as the source of CO_2_.^[^ ^5^, ^6^, ^14^, ^42^ ^]^

**Figure 6:**
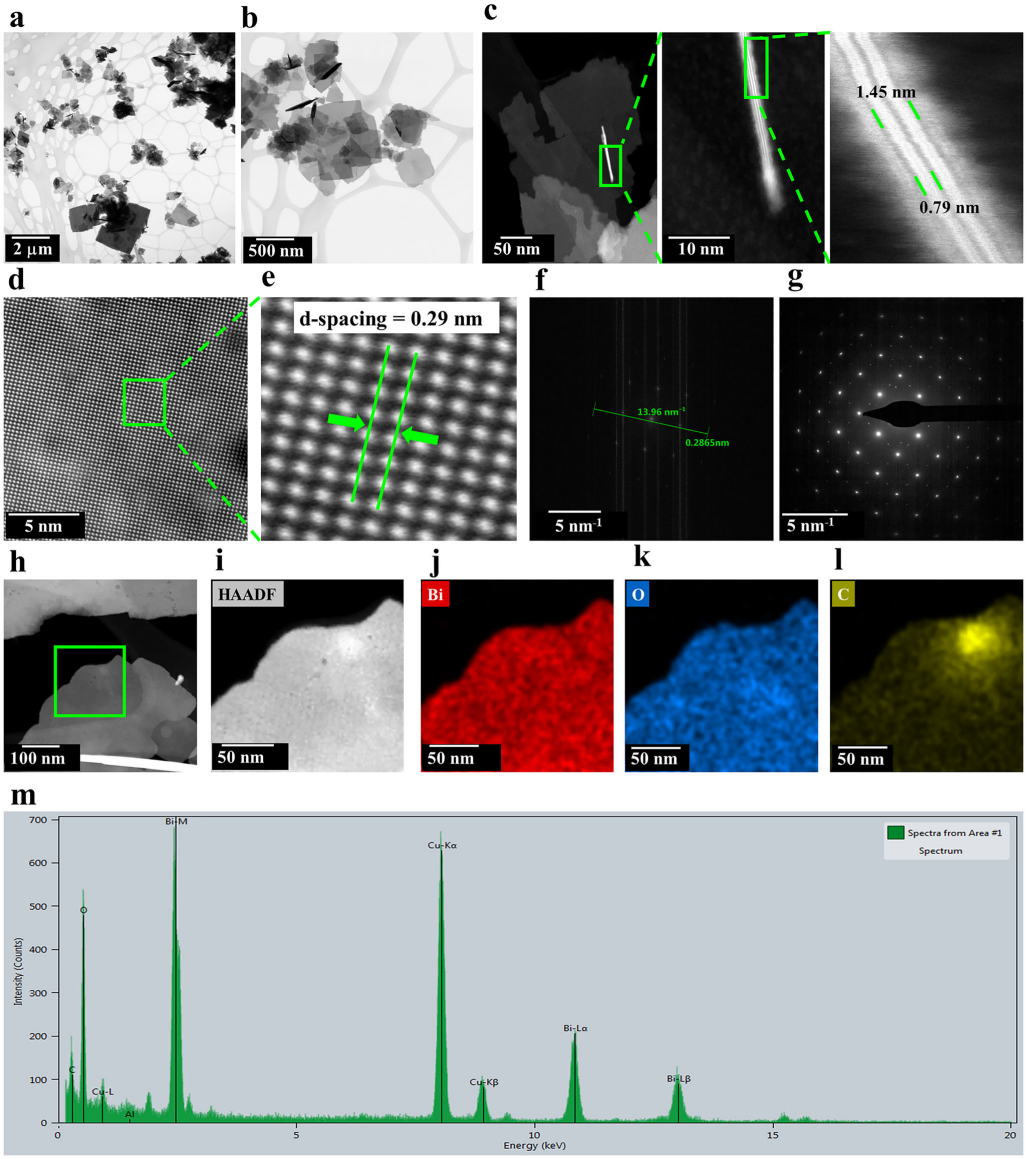
STEM of 2D BOC nanosheets prepared in water at room temperature, 30 minutes processing in the up‐sized VFD, 50 mm OD, *ω* = 2k rpm, *θ* = 45°, confined mode, concentration of Bi 2 mg.mL^−1^, a) and b) Low‐ magnification BF‐STEM images showing the morphology. c) Aberration corrected HAADF‐STEM image of 2D BOC nanosheets. d) HAADF STEM images displaying clear atomic arrangement of 2D BOC nanosheets. e) enlargement of the image shown in (d). f) Fast Fourier transform (FFT) pattern with a measured lattice spacing. g) Selected area electron diffraction (SAED) pattern. h) and i) HAADF‐STEM image of 2D BOC nanosheets. j–l) Elemental maps showing O, Bi, and C, m) EDS spectrum identifying the main elements present.

STEM was employed to examine the size, shape, and the lattice structure of the 2D BOC nanosheets fabricated in the L‐VFD at *ω* = 2k rpm for 30 minutes, *θ* = 45°, and a Bi concentration of 2 mg.mL^−1^. Bright‐field (BF) STEM images shown in (Figure 6a,b) confirm the formation of 2D nanostructure sheets. We note that from previous studies, shear stress in the VFD tube at this rotational speed is equivalent to 5k rpm rotational speed in a standard VFD.^[^ ^28^ ^]^ High‐Angle Annular Dark‐Field (HAADF) STEM image of 2D BOC displayed in Figure 6(c) shows sheets with stacked boundaries and edges which appear in parallel arrays. The results are consistent with Bi─O layers interposed with a layer of CO_3_^2−^, as expected for 2D BOC nanostructure sheets. Figure 6(c) shows images of increasing magnification to illustrate the interlayered CO_3_^2−^ having a 0.79 nm spacing between layers as measured by Fiji ImageJ software. This value is slightly higher than the value of 0.76 nm for other interlayered CO_3_^2−^ structures^[^ ^42^ ^]^ with the large layer distance facilitating CO_2_ capture.^[^ ^42^ ^]^ Figure 6 (d, e) shows HAADF‐STEM images displaying clear atomic arrangement of 2D BOC nanosheets using fast Fourier transform (FFT) pattern, Figure 6 (f); the dotted lines and bright spots represent the periodic arrangement of atoms in the crystal lattice of BOC nanosheets with the green line. The 13.96 nm^−1^ indicates the measured separation between two different spots. This value was used to calculated the lattice spacing d = 0.29 nm for the (103) plane, which corresponds to 2D BOC nanosheets.^[^ ^42^, ^43^ ^]^ The selected area electron diffraction (SAED) pattern, Figure 6 (g), explored the crystallinity of the 2D BOC, which determines the specific crystallographic plane within the crystal lattice, establishing uniformity of the crystal structure. Additionally, Figure 6 (h,i) shows HAADF‐STEM images of the location used for Energy Dispersive X‐ray Spectroscopy (EDS) elemental mapping of 2D BOC. Figures 6 (j–l) shows the elemental mapping conducted using EDS‐STEM to explore the distribution of Bi, oxygen (O), and carbon (C) within the nanosheets. They establish a uniform distribution of these elements, as expected for the presence of uniform 2D BOC sheets. The presence of ultrathin thin sheets of 2D BOC is consistent with SEM images, and thus the material formed in the 20 mm OD VFD is analogous with the material formed in the 50 mm OD VFD. When coupled with Raman spectra results, the formation of high quality crystalline 2D BOC nanosheets is supported.

**Figure 7:**
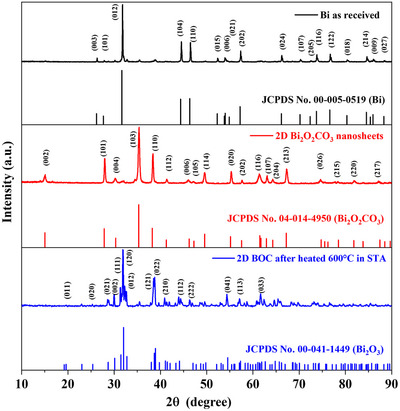
X‐ray diffraction of Bi as received (black line), and 2D BOC nanosheets (red line) fabricated in water at room temperature, 10 minutes processing in a standard VFD tube 20 mm OD, *ω* = 5k rpm, *θ* = 45°, confined mode, concentration 1 mg.mL^−1^, and 2D BOC nanosheets after being heated to 600 °C in STA with the material prepared in water at room temperature, 30 minutes processing in the up‐sized VFD, *ω* = 2k rpm, *θ* = 45°, confined mode, concentration 2 mg.mL^−1^ (blue line).

XRD patterns of Bi as‐received and 2D BOC thin layer sheets were investigated in the range 2*θ* = 10°–90°, as shown in Figure 7 in black and red lines, respectively. This is for material fabricated in the standard VFD 20 mm OD for 10 minutes at *ω* = 5k rpm in the confined mode. XRD patterns of the Bi before VFD processing have a low intensity peak for the α‐phase of Bi_2_O_3_^[^ ^44^ ^]^ at 2*θ* = 30.1°, 32.7°, 35.4°, and 38.8°, presumably as a consequence of the grinding of the metal in an atmosphere of air before VFD processing. The major peak of Bi hexagonal phase at 2*θ* = 31.6° of the (012) plane has the expected d‐spacing of 0.32 nm and the crystallite size (D), as determined from the Scherrer equation, is 76.71 nm, Equation (S1).

The XRD pattern data of 2D BOC sheets confirms the formation of BOC with no evidence for the formation of 2D Bi. This is unlike the VFD processing of antimony, which undergoes a melting/crystallization process to form 2D antimonene.^[^ ^28^ ^]^ The uptake of CO_2_ is from air, thus highlighting high mass transfer of air into the thin film of water in the VFD ^[^ ^5^, ^6^ ^]^ which is a characteristic of VFD processing in general.^[^ ^32^, ^45^ ^]^ The 2D BOC has the orthorhombic phase of the 2D BOC nanosheets, which matches the BOC PDF standard card No. (04–014–4950). A peak at 2*θ* = 31.7° with a low intensity is related to the major peak of α‐Bi_2_O_3_, that is also consistent with Raman spectra data. The major peak of 2D BOC nanosheets in the orthorhombic phase at 2*θ* = 35.3° of (103) with a d‐spacing of 0.29 nm agrees with the STEM data. Also noteworthy is that the crystallite (domain) size (D) of 2D BOC nanosheets calculated by the Scherrer equation is 30.98 nm, Equation (S2).

The decrease of crystallite size during the oxidation of Bi to form BOC is interesting, although the conversion of one crystallite of Bi to BOC is unlikely given the initially likely melting Bi as the primary process which occurs on the inner tube surface at room temperature,^[^ ^19^, ^28^ ^]^ most likely in the confines of the ST flow with the metal rapidly oxidized with then uptake of CO_2_, with the conversion complete in 10 minutes without the need for applying an external heating source. The smaller crystallite size of 2D BOC nanosheets compared to bulk Bi highlights the effect of rapid and localized heating and cooling, limitting crystal growth, which is a phenomenon that also occurred during Sb melting.^[^ ^28^ ^]^

Attempts to obtain meaningful XPS data was not possible due to sample charging effects, with ∼ 5 eV shifts for Bi 4f_7/2_ and Bi 4f_5/2_, at 162.3 eV and 167.6 eV, respectively, Figure S3.

**Figure 8:**
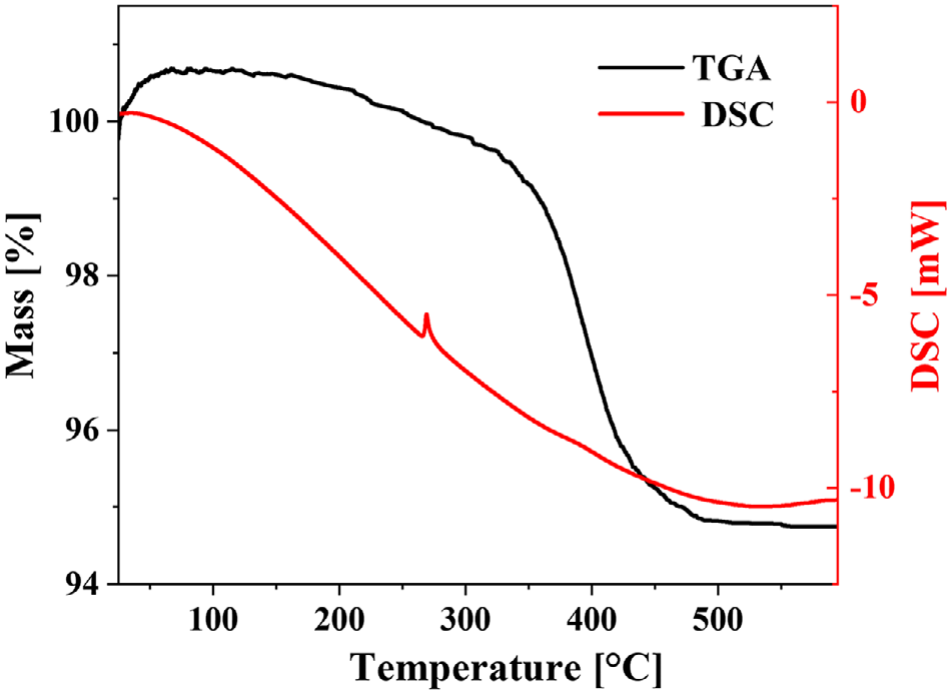
STA shows TGA and DSC curves for 2D BOC nanosheets prepared in water at room temperature, 30 minutes processing in the up‐sized VFD tube 50 mm OD, *ω* = 2k rpm, *θ* = 45°, confined mode, concentration 2 mg.mL^−1^, heated up to 600 °C under N_2_ gas at a flow rate of 10 °C.min^−1^.

The thermal behavior of the 2D BOC nanosheets was investigated using STA which is a combined TGA and DSC technique. As illustrated in Figure 8, 5.0 mg of 2D BOC nanosheets fabricated in the L‐VFD 50 mm quartz tube was heated at a rate of 10 °C.min^−1^ throughout a range of 60 to 600 °C in an N_2_ atmosphere. The TGA shows a total weight loss of 2D BOC nanosheets of 5.25% between 250 and 480 °C.^[^ ^46^ ^]^ This is consistent with the breakdown of 2D BOC, which is typical for carbonate degradation, and results in the conversion of 2D BOC nanosheets into Bi_2_O_3_, a stable material, as shown by the TGA curve stabilizing above 480 to 600 °C. The DSC curve shows an endothermic peak at 269.1 °C with a 15.9 J.g^−1^ area. This peak is related to the growth and conversion of BOC orthorhombic phase into Bi_2_O_3_ in the monoclinic phase, which is consistent with XRD data for BOC after heating to 600 °C, as shown in Figure 7 (blue line).

Following the STA analyses, XRD was conducted to investigate the BOC nanosheets after being heated up to 600 °C. This established the formation of the monoclinic phase of α‐Bi_2_O_3,_ in accordance with PDF standard card (00–041–1449), Figure 7. The XRD pattern of Bi_2_O_3_ (blue line) illustrates that the major high intensity peak at 2*θ* = 31.8° of (120) plane has an expected d‐spacing of 0.32 nm,^[^ ^47^ ^]^ with a mean particle size (D) of Bi_2_O_3_ nanosheets of 44.13 nm, Equation (S3).

## 4 Conclusion

We report a simple, short processing time, high yielding synthesis of orthorhombic 2D BOC nanosheets using VFD processing at room temperature, with the integrity of the product established using a number of techniques, including STEM. The 2D material showed the degradation of the sub‐layer of carbonate into CO_2_ up to 480 °C, forming Bi_2_O_3_ which is stable up to 600 °C. The VFD processing is in the absence of added reagents and in this context the overall processing in water has high green chemistry metrics, for potential scaling up for downstream applications. The findings further highlight the utility of the VFD in making 2D materials, with the mechanism of forming the BOC from elemental Bi distinctly different to other methods of making 2D materials in the device including exfoliation and recrystallization. The novelty of the processing relies on the high mass transfer of atmospheric CO_2_ into the water phase, and this also showcases the VFD as a processing platform for removing CO_2_ from the atmosphere, as a remediation process for anthropogenic buildup of the greenhouse gas. In addition, the findings offer insight into the potential for forming other 2D materials directly from an element where there is a requirement of mass transfer into the liquid under high shear. The ability to transform Bi metal into 2D BOC relied on the specific fluid flow regime in the VFD, with the dominant shear stress required to generate the material being the ST typhoon like flow down to one micron in diameter or less. The energy consumption efficiency of VFD processing coupled with the ability to upscale the process using the larger diameter L‐VFD, augers well for industrial translation of the BOC fabrication and associated applications.^[^ ^48^ ^]^

## Author Contributions

F.A.A. carried out the optimization experiments of synthesis, and analysis date of SEM, XRD, Raman, STEM, and STA studies, and wrote the draft of manuscript, M.A. analyzed, interpreted, and data‐fitted the XPS, A.K.M carried out AFM studies, J.G carried out Raman studies, A.S carried out STEM studies, J.C carried out STA studies. All authors contributed to editing the manuscript and C.L.R. coordinated the research and finalized the manuscript.

## Conflict of Interest

The authors declare no conflict of interest.

## Supporting information



Supporting Information

## Data Availability

The data that support the findings of this study are available in the Supporting Information of this article.
