# Supplementary material for: High Shear Conversion of Elemental Bismuth in Water Under Air to 2D Bismuth Oxycarbonate
Source: Chemistry. 2025 Sep 10;31(58):e02202. doi: 10.1002/chem.202502202 (PMC12531468; doi:10.1002/chem.202502202)
Supplement: Supplementary file 1 — Supporting Information [file CHEM-31-e02202-s001.pdf]

Supplementary information (SI)

High Shear Conversion of Elemental Bismuth in Water Under Air to 2D Bismuth Oxycarbonate

Fayed Abdullah Alrashaidi,[ab] Jason R. Gascooke,[a] Mohammed Z. Asiri,[acd] Abigail K. Mann, [a] Ashley Slattery,[e] Jonathan A. Campbell,[a] Youhong Tang,[a] and Colin L. Raston *[a]

[a] Institute for Nanoscale Science and Technology, College of Science and Engineering, Flinders University, Bedford Park, South Australia, 5042 Australia

[b] Department of Chemistry, College of Science, Jouf University, P.O. Box 2014, Sakaka, 72388, Saudi Arabia

[c] School of Physics, Chemistry and Earth Sciences, University of Adelaide, South Australia 5005, Australia

[d] Physics Department, Prince Sattam Bin Abdulaziz University, Al-Kharj 16278, Saudi Arabia.

[e] Adelaide Microscopy, The University of Adelaide, Adelaide, South Australia 5005, Australia

The crystallite size (D) of Bi as received is calculated by the Scherrer equation:

Equation (S1):

$$\text{D of Bi as received} = \frac{kx\lambda}{(FWHM)\cos\theta} = \frac{0.9x\ 0.179}{0.0022\cos(15.85)} = 76.71\ \text{nm}\ \pm 0.1\ \text{nm}.$$

The crystallite size (D) of 2D BOC nanosheets was calculated by the Scherrer equation:

Equation (S2):

$$\text{D of 2D BOC nanosheets} = \frac{kx\lambda}{(FWHM)\cos\theta} = \frac{0.9x\ 0.179}{0.0055\cos(17.65)} = 30.98\ \text{nm}\ \pm 0.1\ \text{nm}.$$

The crystallite size (D) of 2D BOC nanosheets after heated in STA at 600 ^oC was calculated by the Scherrer equation:

Equation (S3):

$$\text{D of 2D BOC after heated at 600 }^oC\text{ in STA} = \frac{kx\lambda}{(FWHM)\cos\theta} = \frac{0.9x\ 0.179}{0.0038\cos(15.95)}$$

$$= 44.13\ \text{nm}\ \pm 0.1\ \text{nm}.$$

Equation (S4):

$$\% \text{ yield} = \frac{mass\ of\ BOC\ nanosheets}{\text{mass of Bi}} \times 100 = \frac{0.84\ \ mg}{3\text{ mg}} \times 100 = 28\%$$

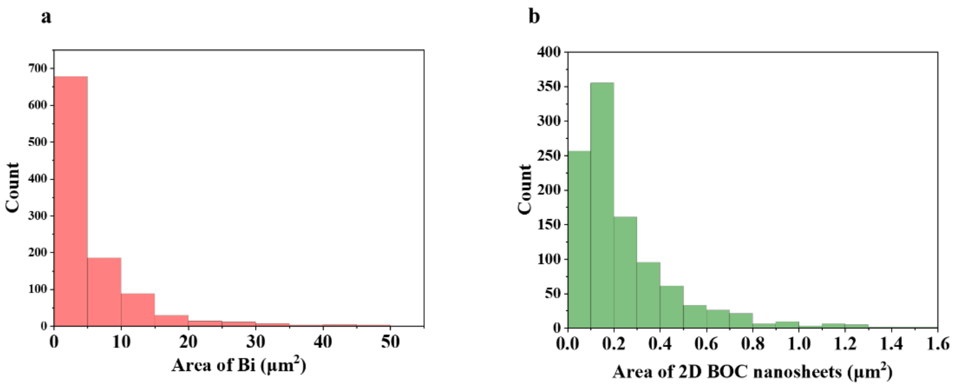

Figure S1. (a) Cross sectional area of particles distribution size of Bi as received, and (b) cross sectional area of particles distribution size of 2D BOC nanosheets.

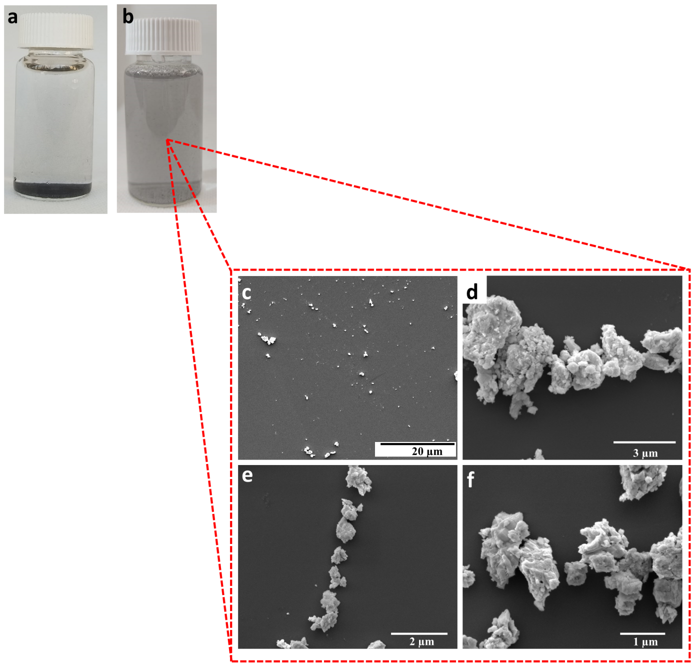

Figure S2. Photographs of dispersed ground Bi powder in Milli-Q water (a) before sonication showing a precipitate at the bottom of the vial, (b) colloidal suspensions in Milli-Q water after sonication of the mixture in (a) for 1 min at 6 kHz, (c-f) SEM images of the material after drop casting and evaporation of colloidal suspensions in Milli-Q water following sonication at 6 kHz for 1 min.

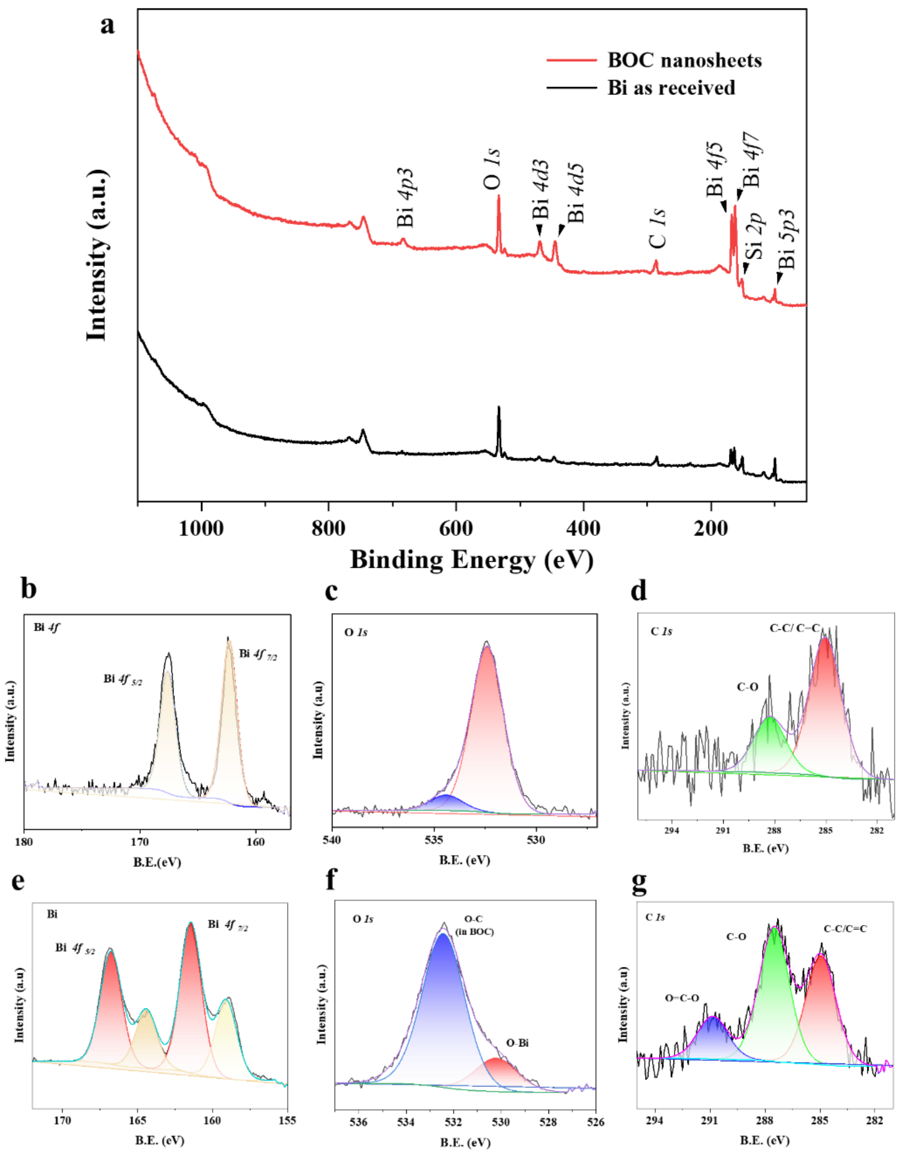

Figure S3. X-ray photoelectron spectroscopy (XPS) spectra of a) Bi as received and 2D BOC nanosheets fabricated in H_2O at room temperature, 10 mins processed in standard VFD 20 mm OD, ω = 5k rpm, θ= 45°, confined mode, concentration 1 $mg.mL^{-1}$, drop casted on Si wafer and dried in an oven at 50 °C. HR-XPS of Bi as received (b) Bi 4f, (c) O 1s, (d) C 1s. HR-XPS of 2D BOC nanosheets (e) Bi 4f, (f) O 1s, and (g) C 1s.
